# Integrating Treatment for Co-Occurring Mental Health Conditions

**DOI:** 10.35946/arcr.v40.1.07

**Published:** 2019-10-24

**Authors:** Amy M. Yule, John F. Kelly

**Affiliations:** **Amy M. Yule, M.D**., is a psychiatrist at Massachusetts General Hospital and an assistant professor of psychiatry at Harvard Medical School, Boston, Massachusetts. **John F. Kelly, Ph.D**., is a psychologist at Massachusetts General Hospital and a professor of psychiatry at Harvard Medical School, Boston, Massachusetts

**Keywords:** alcohol use disorder, integrated treatment, mental health condition, screening, treatment setting

## Abstract

Given the high co-occurrence between alcohol use disorder (AUD) and mental health conditions (MHCs), and the increased morbidity associated with the presence of co-occurring disorders, it is important that co-occurring disorders be identified and both disorders addressed in integrated treatment. Tremendous heterogeneity exists among individuals with co-occurring conditions, and factors related to both AUD and MHCs, including symptom type and acuity, illness severity, the chronicity of symptoms, and recovery capital, should be considered when recommending treatment interventions. This article reviews the prevalence of co-occurring AUD and MHCs, screening tools to identify individuals with symptoms of AUD and MHCs, and subsequent assessment of co-occurring disorders. Types of integrated treatment and current challenges to integrate treatment for co-occurring disorders effectively are reviewed. Innovative uses of technology to improve education on co-occurring disorders and treatment delivery are also discussed. Systemic challenges exist to providing integrated treatment in all treatment settings, and continued research is needed to determine ways to improve access to treatment.

## Introduction

Given the high co-occurrence between alcohol use disorder (AUD) and mental health conditions (MHCs),^1^ and the increased morbidity associated with the presence of co-occurring disorders,^2^ it is important to identify the co-occurring disorders and to address both disorders in treatment to improve treatment outcome. Treatment that addresses both disorders concurrently with the same provider or treatment team is called integrated treatment. As integrated treatments continue to be developed, evaluated, and implemented, the heterogeneity associated with co-occurring AUD and MHCs needs to be acknowledged, since it can affect individual functioning and prognosis. Factors that contribute to heterogeneity among individuals with co-occurring AUD and MHCs include acuity of symptoms, severity of illness, chronicity of symptoms, co-occurring drug use, physical health, cognitive impairment, and recovery capital (Table 1). Recovery capital is a newer dimension to consider, which includes the amount of available resources a person has to support stabilization of AUD and the transition into recovery.^3^

This article provides a background on the prevalence of AUD and co-occurring MHCs, discusses screening tools to identify individuals with symptoms of problematic alcohol use and an MHC, and discusses subsequent assessment of co-occurring disorders. Patient placement considerations and types of integrated treatment are also covered. The article concludes with a discussion of the challenges of integrating treatment for co-occurring disorders effectively and the recent innovations in education and treatment delivery that address some of these challenges.

## Background

Over the past 30 years, there has been increasing awareness that AUD frequently co-occurs with MHCs. The high rate of co-occurring AUD and MHCs is not surprising, since research has demonstrated that young people with a history of an MHC, when compared to peers with no MHC history, are at increased risk to initiate alcohol use, transition to regular use, and subsequently develop AUD.^4^ Furthermore, co-occurrence begins to emerge early. One study found that adolescents with an MHC had onset of alcohol use, regular alcohol use, and AUD at median ages of 12.2 years, 13.8 years, and 14.3 years, respectively.^4^

Individuals with AUD, when compared to individuals with MHCs, have a higher prevalence of co-occurring disorders. More specifically, among adults in the United States in 2017, an estimated 14.1 million had AUD, and 46.6 million had an MHC.^1^ Within these two groups, 5.9 million adults had current, co-occurring AUD and MHCs, which represents 41.8% of individuals with current AUD and 12.7% of individuals with a current MHC. In adults, AUD has been associated with an increased lifetime risk for major depressive disorder (adjusted *OR* of 1.3), anxiety disorder (adjusted *OR* of 1.3), and bipolar I disorder (adjusted *OR* of 2.0), as well as with antisocial and borderline personality disorders (adjusted *OR*s of 1.9 and 2.0, respectively).^5^ For MHCs, a history of childhood attention deficit hyperactivity disorder, oppositional defiant disorder, or conduct disorder has been associated with an increased risk for developing AUD,^6^ and bipolar I disorder, antisocial personality disorder, and psychotic spectrum illness have been associated with substantially higher rates of lifetime and current AUD.^7^,^8^

Co-occurring AUD and MHCs have been associated with poorer outcomes, such as increased rate of relapse,^9^ use of psychiatric services, and use of emergency services,^2^ when compared to each disorder separately. Although treatment interventions have been developed specifically for individuals with AUD, most treatment is provided in clinical settings that treat both AUD and other drug use disorders, hereafter called substance use disorder (SUD) treatment.

Until the increased recognition of co-occurring disorders in the 1980s and 1990s, patients who presented for SUD or mental health treatment often were not evaluated for a co-occurring disorder, or their treatment plan did not address the co-occurring disorder. Since neither disorder is likely to show sustained improvement if one disorder is treated without acknowledging the presence or influence of the co-occurring disorder,^10^–^13^ different treatment approaches were developed to address co-occurrence, including sequential, parallel, and integrated treatments. In sequential treatment, one disorder is assessed and treated before addressing the other disorder. In parallel treatment, different providers or treatment teams address each disorder separately. In integrated treatment, the same provider or treatment team addresses both disorders concurrently.

If one treatment team provides care, the providers work in the same setting and coordinate care. Colocation of treatment and coordinated care helps providers give patients a consistent message regarding treatment and recovery.^14^ Integrated treatment is considered the standard of care regardless of the treatment setting (SUD or mental health) a patient presents to first.^15^

To support the dissemination of integrated treatment, the Substance Abuse and Mental Health Services Administration (SAMHSA) released the Integrated Treatment for Co-Occurring Disorders Evidence-Based Practices Kit in 2009, which remains publicly available.^16^ Since then, SAMHSA and the Health Resources and Services Administration established a Center for Integrated Health Solutions to support the development of integrated primary and behavioral health care for MHCs, SUD, and physical health conditions such as hypertension, obesity, and cardiovascular disease. These efforts are needed, since most individuals with co-occurring SUD and MHCs do not receive integrated treatment. For example, in 2017, only 8.3% of adults with an MHC and co-occurring SUD received mental health and SUD services, whereas 38.2% received mental health services only, 4.4% received SUD treatment only, and 49% received no treatment.^1^

## Screening and Assessment

One factor contributing to low rates of integrated treatment for individuals with co-occurring AUD and MHCs is poor identification of the presence of a co-occurring disorder. Like other health conditions for which routine screening occurs at certain ages (e.g., breast cancer screening for women beginning at age 40) or in certain settings (e.g., screening for hyperlipidemia in primary care settings), screening for both the presence of AUD and for other MHCs can be efficiently conducted. This screening, however, may be rare in practice, especially among certain subgroups. One review found that adolescents, individuals from low socioeconomic backgrounds, and racial/ethnic minorities often are not identified as having a co-occurring disorder, despite having both disorders.^17^ Routine, standardized screening is necessary to identify problematic alcohol use and mental health symptoms and to assess for co-occurring disorders.

Screening for alcohol and other substance use in the medical setting has become the standard of care because of the demonstrated efficacy of screening, brief intervention, and referral to treatment (SBIRT) in the primary care setting for reducing problematic alcohol use.^18^ Over the past 15 years, emphasis on implementing SBIRT in other health care settings, such as emergency departments and inpatient medical settings, has increased.^19^ Given the relationship between AUD and MHCs, these medical settings present opportunities for incorporating screening for mental health symptoms with screening for problematic alcohol use, and further research is needed on how to do this. Likewise, more research is needed on the effectiveness of SBIRT in the mental health treatment setting, since most individuals with co-occurring MHCs and AUD receive mental health treatment only. Table 2 lists representative examples of screening tools that assess for problematic alcohol use and other substance use. Screening for symptoms of an MHC in an SUD treatment setting is also necessary. Table 3 includes examples of screening tools for MHCs.

In addition to detecting the presence or absence of co-occurring AUD or MHCs, understanding the nature, scope, chronicity, and effect of the primary disorder and the co-occurring ones is critically important for formulating an effective treatment and recovery plan. Typically, this process is called the assessment, in contradistinction to the initial screening. Longer comprehensive assessment tools for SUD that also assess for problems related to an MHC have been used in clinical trials and in the community. These tools include the semistructured Addiction Severity Index (ASI),^31^ the Global Appraisal of Individual Needs (GAIN),^32^ and the American Society of Addiction Medicine (ASAM) Criteria.^33^ The psychiatric scales from the ASI have been shown to be an effective tool for identifying individuals with a co-occurring MHC, but further assessment is needed to determine which co-occurring disorder is present.^34^ The GAIN assesses for symptoms of specific psychiatric disorders, including internalizing disorders such as depression, anxiety, trauma, and suicide, as well as externalizing disorders such as attention deficit hyperactivity disorder and conduct disorder.^32^ The ASAM Criteria was designed to help clinicians determine the recommended treatment setting and level of care for patients with SUD, but it includes a brief mental health symptom assessment that can be used to identify acute psychiatric safety concerns and symptoms that need further assessment.^33^

One challenge to screening and assessing for co-occurring MHCs in individuals with AUD is that problematic alcohol use is associated with changes in mood, sleep, concentration, and anxiety. Initially, it may be unclear if someone suffers from a co-occurring MHC that is independent of alcohol or drug use and that warrants focused attention, or if symptoms or the apparent disorder will dissipate with alcohol or drug abstinence. To address this challenge, the fifth edition of the *Diagnostic and Statistical Manual of Mental Disorders* (DSM) includes the diagnosis “alcohol-induced mental disorders” to describe symptoms of a temporary MHC only observed during severe alcohol intoxication or during withdrawal from alcohol.^35^ Therefore, comprehensive screening and assessment of co-occurring MHCs should not be done when an individual is intoxicated or is experiencing withdrawal symptoms. Generally, in addition to screening for symptoms of an MHC during an individual’s initial engagement in treatment, clinicians should reassess mental health symptoms later during treatment to confirm the diagnosis and severity of the MHC and to plan for treatment.

Although there should be no “wrong door” for treatment when an individual with AUD and a co-occurring MHC presents for care, until integrated treatment of both disorders is more commonplace, clinicians need to consider the severity and effects of each disorder when recommending treatment settings. The quadrant model is a tool that can be used to help clinicians make these recommendations. The quadrant model has four treatment categories based on the severity of the SUD and MHC: the primary health care setting, the SUD setting, the mental health system, and specialized co-occurring disorder programs.^36^ This model has been adopted by national addiction and mental health treatment administrators,^37^ has been validated as effective at categorizing patients with co-occurring disorders, and has been associated with appropriate service utilization.^38^

The quadrant model can also help clinicians assess whether a patient would benefit from referral to a different treatment program to expedite symptom stabilization and maximize treatment efficacy. However, the quadrant model assumes comprehensive screening and assessment of substance use and mental health symptoms. Thus, continued efforts are needed to improve screening for both disorders to facilitate a thorough assessment and subsequent referral to appropriate treatment. Most patients and families do not know or understand the differences between treatment settings, so more research is needed on how to facilitate treatment referrals so patients remain engaged in care.

## Types of Integrated Treatment

Regardless of the treatment setting, behavioral therapy, pharmacotherapy, and recovery support in the patient’s community should be considered in treatment plans for patients with co-occurring AUD and MHCs. Because of the heterogeneity among co-occurring AUD and MHCs, individualized treatment plans should account for the severity of each disorder and for patient preference regarding interventions. Also, although not typically assessed, the amount of available resources a person has for stabilization and recovery needs to be included in the assessment to inform the treatment plan. These resources often are called “recovery capital,” a dimension^3^ that recently developed tools can assess.^39^,^40^ Two clinically identical patients can have different levels of recovery capital in terms of employment, education, finances, living situation, and social networks, all of which can affect clinical interventions and, ultimately, the likelihood of remission and long-term recovery.

### Behavioral therapy

Behavioral therapies, such as motivational enhancement therapy, cognitive behavioral therapy, contingency management, and 12-step facilitation, are the standard of care for individuals with AUD and are a key part of a treatment plan for individuals with co-occurring AUD and MHCs.^41^ As such, behavioral therapy for AUD, which is commonly motivational enhancement therapy or cognitive behavioral therapy, is provided to all participants in most randomized controlled trials that evaluate pharmacotherapy for individuals with AUD and an MHC. Although less commonly discussed, AUD-focused therapies delivered to individuals with MHCs may need to be adapted to account for the MHC. For example, Levin and colleagues modified the delivery of cognitive behavioral therapy for SUD when working with individuals who had co-occurring attention deficit hyperactivity disorder.^42^ The researchers allowed in-session time for completing homework assignments, checked in with participants after presenting any new paradigm for understanding drug use behavior, and used visual diagrams to help with skills training.

Other behavioral therapies designed to address MHCs, such as cognitive behavioral therapy for depression or anxiety and dialectical behavioral therapy for mood dysregulation, can be integrated into the treatment plan for individuals who have co-occurring disorders. For example, integration of modules from cognitive behavioral therapy for individuals with AUD and depression may include introducing skills to address each disorder at alternating sessions. Increasingly, co-occurring disorders are being addressed simultaneously in a single session. Examples include integrated group therapy for adults with bipolar disorder and SUD,^43^ integrated individual cognitive behavioral therapy for depression and SUD,^44^ integrated cognitive behavioral therapy for post-traumatic stress disorder and SUD,^45^ and “seeking safety,” a group therapy for individuals with a history of trauma and SUD.^46^

These integrated protocols appear to be promising. Researchers that conducted a meta-analysis of studies that combined cognitive behavioral therapy and motivation interviewing to treat individuals with depression and AUD found that integrated treatment, when compared to usual care, was associated with small but clinically significant improvements in depressive symptoms and alcohol use.^47^ Another review of integrated treatments for individuals with SUD and trauma experiences also found that integrated treatment was associated with improvement in both SUD and symptoms of post-traumatic stress disorder, but no clear benefit was found for integrated treatment when it was compared to nonintegrated treatment.^48^ Further research is needed to compare the efficacy, cost, and patient satisfaction associated with integrated versus nonintegrated behavioral treatment of AUD and MHCs.

### Pharmacotherapy

Pharmacologic trials for co-occurring AUD and MHCs have focused primarily on treating the MHC with a medication that has demonstrated efficacy for treating the MHC in the absence of co-occurring AUD.^49^–^51^ This type of trial includes, for example, using an antidepressant medication to treat an individual who has AUD and major depressive disorder. On average, these pharmacologic trials have shown modest improvements in the MHC, with limited improvement in the co-occurring AUD.^52^,^53^ Likewise, clinical trials that used medication effective at treating AUD alone have shown some improvement in the AUD, with limited improvement in the co-occurring MHC.^50^,^54^ Importantly, in the studies that evaluated the effectiveness of AUD medication for co-occurring AUD and MHCs, most participants were also simultaneously receiving medication for the MHC, which may have affected study outcome.^54^,^55^

Pharmacologic trials for co-occurring disorders have been limited by small sample sizes, which reflects difficulty recruiting and retaining participants in these trials. Given these challenges, studies using registries or electronic medical record databases may be an alternative for evaluating outcomes associated with available pharmacologic treatments. For example, one recent quasi-experimental study used public databases to examine the effect of medication treatment for AUD among adults involved in the criminal justice system.^56^ These participants had alcohol dependence (per the DSM-IV classification) and serious mental illness (i.e., schizophrenia, bipolar disorder, or major depressive disorder). Although details on abstinence, heavy-drinking days, and symptoms of the MHC were not accessible through the public databases used in this study, the databases allowed investigators to identify a large sample (*N* = 5,743) and use information on functional outcomes, which served as a proxy for traditional outcomes used in a randomized controlled trial. In this study, individuals who received medication for AUD were less likely at the 1-year follow-up to have been hospitalized for a psychiatric condition or to have used the emergency department. They also were more likely to have adhered to their psychotropic medication regimen than participants who were not taking these medications.

The overall literature on pharmacotherapy for co-occurring AUD and MHCs suggests medication without other treatment interventions may not be adequate to stabilize both conditions.^52^,^57^ Nonetheless, medication is a treatment option that should be discussed with patients who have co-occurring disorders. For more serious mental illness, specifically bipolar disorder and psychotic disorders, disorder-specific medication is necessary for initial stabilization and maintenance.^37^ For other MHCs, such as depression and anxiety with mild to moderate impairment and AUD with mild impairment, when each disorder is considered separately, treatment guidelines suggest medication or therapy as options for first-line treatment, although medication is more strongly indicated for individuals who have greater impairment.^58^–^60^ More research is needed to determine if medication should be more strongly indicated for co-occurring AUD and MHCs causing mild impairment, given the more complicated course of illness when these disorders co-occur.

### Recovery support in the community

Peer-led mutual help organizations can be another component of a treatment plan for individuals with co-occurring AUD and MHCs. Beginning in the 1980s, mutual help organizations for individuals with SUD and an MHC were formed, including Dual Recovery Anonymous, Double Trouble in Recovery, and Dual Diagnosis Anonymous.^61^ These groups all follow the 12 phases or traditions of 12-step organizations, but they have modifications addressing the co-occurring MHC. Relative to 12-step organizations for AUD alone, such as Alcoholics Anonymous, mutual help groups for individuals with co-occurring disorders are less common, and less research exists that evaluates the relationships among group attendance, mental health symptoms, and alcohol use. In one study of individuals with psychotic disorders (schizophrenia or schizoaffective disorder) and AUD and/or cocaine use disorder, in which a majority of the participants were African American, investigators found that regular attendance at Double Trouble in Recovery was associated with fewer psychiatric symptoms, increased rates of abstinence, and greater adherence to psychiatric medication.^62^

Because of their greater national presence, mutual help organizations for AUD or MHCs are much more accessible than those for co-occurring disorders. Among the mutual help organizations for AUD, Alcoholics Anonymous is the largest, with approximately 61,000 meetings serving 1.3 million members in the United States.^63^ Also, Alcoholics Anonymous has been the mutual help organization most thoroughly evaluated for the effect of participation, both for individuals with AUD and for those with co-occurring AUD and an MHC. A recent systematic review and meta-analysis of patients with AUD and co-occurring MHCs found that AUD improved with Alcoholics Anonymous attendance, and the patients with co-occurring AUD and an MHC benefited from engagement with Alcoholics Anonymous as much as patients with no co-occurring MHC.^64^

Mutual help organizations for individuals with MHCs have greatly expanded over the past 30 years as part of an overall emphasis on including peers in the recovery process. Whether participation in these groups provides benefit has been less clear,^65^ and research in this area has been complicated by a lack of standardization across groups. Substantial variability exists regarding services provided by these groups, which can include telephone support hotlines, social and recreational activities, and advocacy, in addition to face-to-face meetings. Also, research evaluating the efficacy of these groups has not examined differences between individuals who have an MHC with a co-occurring AUD and those with no co-occurring AUD. Further research is needed to determine the ways individuals with co-occurring AUD and MHCs might benefit from participation in a mutual help organization that is focused on alcohol and other substance use versus a group focused on symptoms of the MHC.

In addition to in-person peer support, individuals who have AUD and/or MHCs are increasingly seeking support through online support groups and social media.^66^,^67^ Research is ongoing to determine the effectiveness, important characteristics (e.g., synchronous, such as chat rooms; asynchronous, such as forums; and level of monitoring from moderators), and risks of online peer support. Because of the heterogeneity associated with co-occurring AUD and MHCs, people with similar illness experiences may be geographically far apart, and online peer support could help them connect.

### Comprehensive integrated treatment for serious mental illness and AUD

Evidence-based practices for integrated treatment programs for individuals with substantial impairment and low functioning because of AUD and a serious mental illness, such as schizophrenia or bipolar disorder, include incorporating interventions that match an individual’s stage of readiness for treatment engagement^68^ and involve assertive outreach, motivational interventions, and counseling to build cognitive and behavioral skills. Evidence-based practices also include strengthening an individual’s connection with social supports that encourage recovery, a comprehensive approach that addresses AUD and MHCs in all aspects of the program, including social services, and takes a long-term, community-based perspective on recovery. Cultural sensitivity and competence are also crucial aspects of integrated treatment programs.

One example of a comprehensive integrated treatment is integrated dual diagnosis treatment, which incorporates these evidence-based practices and integrates all components of a treatment plan, including psychological, pharmacological, educational, and social interventions.^69^ Assertive community training and intensive case management are two other treatments that have been adapted for individuals with serious mental illness and co-occurring AUD.^37^ These two treatments both involve intensive case management, skills training, and individual counseling.

The research supporting superior efficacy associated with integrated treatment remains limited. However, in a systematic review of randomized controlled trials of long-term integrated psychosocial interventions for individuals with SUD and serious mental illness, when the researchers compared integrated intervention with usual care, they found no significant differences in participant alcohol or substance use, functioning, or life satisfaction.^70^ The investigators noted that their systematic reviews of the existing literature were limited by differences in study design and the outcomes used to evaluate intervention efficacy, as well as by low rates of subject retention, longitudinally.

## Challenges in Implementing Integrated Treatment

Although integrated treatment is considered the standard of care for individuals with co-occurring AUD and MHCs, implementing it in both SUD and mental health treatment centers has been difficult. Some of the implementation challenges relate to the independent development of the public mental health and SUD treatment systems, which have differences in workforce training (e.g., coursework and clinical rotations), licensure requirements, and reimbursement.

Training and licensure requirements for providers delivering the same type of treatment vary among specialties. For example, behavioral therapies are commonly delivered by psychologists, social workers, counselors with primary training in MHCs, or alcohol and drug counselors. The programs that train these providers have different accreditation bodies that oversee the educational requirements during training. The programs also have different state licensure requirements. In 2009, the Council for Accreditation of Counseling and Related Educational Programs revised its standards to emphasize that mental health counselors need to have exposure to coursework specific to substance use.^71^ When mental health counseling programs were surveyed in 2013, 69% required this coursework, and 13% offered it as an elective.^72^ In contrast, the Council on Social Work Education has no emphasis on coursework specific to substance use, and the same survey found only 2% of master’s degree programs in social work required this coursework, and only 64% offered it as an elective.

For alcohol and drug counselors, training traditionally has emphasized clinical rotations, but more recently it has been shifting toward incorporating more formalized coursework.^73^ Unlike other behavioral therapy providers, alcohol and drug counselors have no national accreditation system to guide their training for MHCs, and training programs are more influenced by state licensure requirements. Differences in training and licensure may affect the dissemination and implementation of newer evidence-based practices, such as integrated treatments. Standardized training and licensure requirements could provide a mechanism for monitoring training, and it could potentially encourage dissemination of newer practices through continuing education requirements.

However, requiring that all providers receive training in both SUD and MHCs does not guarantee they will receive didactic and clinical training in both conditions or training in integrated treatment. Training experiences for these disorders generally occur separately. In part, separate training experiences occur because integrated services may not have been developed to serve as a clinical training site, and because many educators lack training and expertise in the management of co-occurring disorders.

For example, although graduate medical education for psychiatry requires that trainees be exposed to addiction psychiatry, concerns have been raised that the current training does not produce psychiatrists who are well-prepared to manage SUD, or co-occurring SUD and MHCs, in practice.^74^ When training directors of general psychiatry were surveyed to identify barriers to adequate training in addiction, the two most commonly identified barriers were limited faculty and staff with expertise, and limited faculty and staff time to supervise clinical experiences.^74^ This survey also found that in 2017, only 15% of general psychiatry training programs had board-certified faculty in addiction psychiatry, and only 37% of programs had board-certified faculty in addiction medicine.

Since no formal training paths offer training in integrated treatment, providers generally need to pursue training in each field to be prepared to provide this type of care. Few incentives exist for pursuing additional training, because within the SUD and mental health treatment systems, additional reimbursement is not provided for delivering integrated treatment services. Reimbursement inequities also exist for each type of care. Historically, insurance benefits for mental health treatment have been greater than the benefits for substance use treatment.^75^

The federal Mental Health Parity and Addiction Equity Act of 2008 was enacted to address this inequity. Despite the legislation, integrated treatment delivery is still limited by restrictive diagnostic and billing criteria that generally assess service eligibility based on one disorder only.^76^ Often, the criteria do not account for the complexity added to either disorder when a co-occurring disorder is present. Furthermore, integrated care often requires frequent communication among providers to effectively coordinate care, but coordination of care is not a reimbursable service in fee-for-service insurance models. SAMHSA continues to work to address these barriers, and it is possible that as health care financing transitions from fee-for-service to population-based care, funding to support integrated treatment programs may become more flexible.

## Innovative Models

One example of an innovative model for improving education is the Extension for Community Healthcare Outcomes program for primary care providers, called Project ECHO (https://echo.unm.edu). This program uses a simultaneous video link to connect specialists and primary care providers in different regions of a state for regular case-based discussions. In New Mexico, one focus of Project ECHO has been a weekly meeting about addictions and psychiatry. A review of the program suggests that this type of learning opportunity helped New Mexico increase the number of physicians who have waivers to prescribe buprenorphine in underserved areas at a much faster rate relative to other states in the country.^77^

Innovative models also have been developed to address some of the challenges associated with implementing integrated treatment, particularly the shortage of providers in the addiction treatment setting who are trained in both SUD and MHCs. When two transdiagnostic and not disorder-specific interventions for MHCs were evaluated among individuals with AUD and co-occurring anxiety disorders, the interventions showed encouraging preliminary results.^78^,^79^

Unified protocol therapy is an emotion-focused, cognitive behavioral therapy treatment that has been shown to be effective for a range of different MHCs, including anxiety, depression, and bipolar disorder. In an 11-week study, 81 individuals who had AUD and an anxiety disorder were randomized to 4 conditions, and the group that received the unified protocol therapy was the only group to have a significant reduction in heavy-drinking days when compared to the other groups.^78^

Acceptance and commitment therapy is a mindfulness-based form of behavioral therapy that has been shown to be effective for anxiety and depression, as well as for SUD. In a 12-week, uncontrolled pilot study of acceptance and commitment therapy, which included 43 veterans with AUD and post-traumatic stress disorder, researchers found that 67% of the veterans completed the protocol.^79^ Improvements in alcohol use, anxiety, depression, and quality of life were also reported. More research is needed to evaluate the efficacy of these transdiagnostic interventions for co-occurring AUD and MHCs. Currently, five clinical trials registered on clinicaltrials.gov are investigating these two transdiagnostic interventions for co-occurring disorders.

Another strategy for addressing implementation challenges has been to leverage technology to help providers who have no prior specialized training deliver cognitive behavioral therapy for anxiety disorders. For example, in the coordinated anxiety learning and management (CALM) intervention for addiction recovery, individuals with SUD and an anxiety disorder receive a group-based, computer-assisted, but therapist-directed, treatment for anxiety disorders that has been adapted for individuals with co-occurring disorders. In a randomized controlled trial, individuals who received the CALM intervention had less anxiety and less substance use through 6-month follow-up when compared to those who received the usual care.^80^

## Future Directions

Although integrated treatment for co-occurring AUD and MHCs makes intuitive sense, the evidence base supporting integrated treatment, particularly for co-occurring anxiety and depression, is less mature. To address the heterogeneity among individuals with co-occurring disorders, more research is needed on the types of services, service providers, and treatment settings that are best for which groups of individuals. Also, in the evaluation of a treatment’s efficacy, it is important to include individual strengths, such as recovery capital, that may moderate or mediate response to treatment. Recruiting participants who have AUD and MHCs for randomized controlled trials to evaluate the effectiveness of treatment can be challenging, and increasing measurement-based practice^81^ within current treatment structures could help clinicians determine which patients are struggling and prompt re-evaluation of treatment plans.

Furthermore, a limited amount of staff and faculty with expertise in integrated treatment for individuals with SUD and MHCs has been identified as a barrier to improving education and subsequent delivery of care for co-occurring disorders. Therefore, it is imperative that educators and policy makers consider increasing virtual and multidisciplinary training opportunities that focus on addiction, MHCs, and integrated treatment. Increasing multidisciplinary training opportunities includes streamlining continuing education accreditation so an educational program developed for one group of providers can easily be shared with other providers who could benefit from the same information and who also need continuing education credits for their specialty.^81^

Finally, continued innovation is needed to use promising technologies, such as computerized interventions, to treat co-occurring disorders in settings that have limited expertise. Although some preliminary projects have evaluated adapting computerized interventions for MHCs for delivery in the SUD treatment setting, no trials of computerized interventions for SUD have been adapted for delivery in the mental health treatment setting. Since most individuals with co-occurring SUD and MHCs receive care in the mental health setting, this is an important setting for evaluating these types of interventions.

## Figures and Tables

**Table 1:** Factors That Affect Functioning and Prognosis for Individuals With Co-Occurring AUD and MHCs

Factor	Examples
Acuity of Symptoms	Symptoms of alcohol withdrawal that require urgent medical management Active suicidal ideation that requires inpatient psychiatric admission Current symptoms of disorder only Lifetime history of disorder
Severity of Illness	Severe AUD Serious mental illness: schizophrenia, bipolar disorder, treatment-resistant major depressive disorder, or anxiety associated with agoraphobia
Chronicity of Symptoms	Recent onset of symptoms Chronic symptoms with minimal periods of recovery
Co-Occurring Drug Use	Injection drug use Substances (e.g., cocaine) associated with psychiatric symptoms (e.g., anxiety and psychosis)
Physical Health	Malnutrition or liver cirrhosis related to chronic alcohol use Physical disability Infectious disease: HIV or hepatitis C Pregnancy and family planning
Cognitive Impairment	Substance related Low IQ Head trauma
Recovery Capital	Employment Education Finances Living situation Social networks

**Table 2:** AUD and SUD Screening and Assessment Tools for the Primary Care Setting

Tool	Description
AUD	
Alcohol Screening and Brief Intervention for Youth: A Practitioner’s Guide^20^	Clinician-administered screening Developed for youth ages 9 to 18 Two questions about patient and peer alcohol use Developmentally specific questions for patients in elementary school, middle school, and high school
Alcohol Use Disorders Identification Test (AUDIT)^21^	Clinician- or patient-administered screening Developed for adults Ten questions about alcohol use, three questions in abbreviated version (AUDIT-C)
AUD and SUD	
Screening to Brief Intervention (S2BI)^22^ Brief Screener for Tobacco, Alcohol, and Other Drugs (BSTAD)^23^	Clinician- or patient-administered screening Developed for adolescents Three initial questions about tobacco, alcohol, and marijuana use in the past year Four additional questions about other types of drugs if adolescent replied yes to any of the three initial questions For S2BI, four choices for frequency of use over the past year For BSTAD, number of days of use over the past year
Tobacco, Alcohol, Prescription Medication, and Other Substance Use (TAPS)^24^	Clinician- or patient-administered screening and assessment Developed for adults Four initial questions about tobacco, alcohol, illicit drugs, and nonmedical use of prescription drugs in the past year Additional questions to assess risk level if patient replied yes to initial questions
National Institute on Drug Abuse (NIDA) Quick Screen^25^	Clinician-administered screening and assessment Developed for adults Four initial questions about frequency of tobacco, alcohol, illicit drug, and nonmedical prescription drug use in the past year Clinician intervention guided by patient response
Alcohol, Smoking and Substance Involvement Screening Test (ASSIST)^26^	Clinician-administered screening and assessment Developed for adults Questions about lifetime and past 3-month use of tobacco, alcohol, and seven other drugs Assessment of frequency, desire to use, and associated substance use problems if patient endorsed substance use in the past 3 months Questions about injection drug use, concern from friends or relatives, and difficulty with decreasing substance use if patient endorsed lifetime substance use

**Table 3:** MHC Screening Tools

Screening Tool	Description
Pediatric Symptom Checklist (PSC)^27^	Parent- or child-administered screening for emotional or behavioral problems Developed for children and adolescents ages 6 to 16 seen in primary care Seventeen or 35 questions that assess psychosocial functioning
Patient Health Questionnaire (PHQ-9)^28^	Patient-administered screening for depression Developed for adults seen in primary care Nine questions
Generalized Anxiety Disorder (GAD-7)^29^	Patient-administered screening for generalized anxiety disorder Developed for adults seen in primary care Seven questions
Mental Health Screening Form III^30^	Clinician- or patient-administered screening to identify psychiatric co-occurrence Developed for adults receiving treatment for SUD Eighteen questions
